# Understanding the Purchase Decisions of Silver Consumers in Short-Form Video Platforms from the Perspective of Existence, Relatedness, and Growth Needs

**DOI:** 10.3390/bs13121011

**Published:** 2023-12-13

**Authors:** Xicheng Yin, Yicheng Li, Rui Gao, Jieqiong Li, Hongwei Wang

**Affiliations:** 1 School of Cyber Science and Engineering, Nanjing University of Science and Technology, Nanjing 210014, China; xichengyin@njust.edu.cn (X.Y.);; 2 School of Economics and Management, Tongji University, Shanghai 200092, China; hwwang@tongji.edu.cn

**Keywords:** elderly consumer, short-form video, purchase intension, existence, relatedness, and growth (ERG) theory

## Abstract

The differentiated characteristics of the silver (elderly) group from other groups means that the previous interaction mechanism in short-form video (SFV) e-commerce is no longer applicable. Drawing on sociotechnical systems theory and the ERG theory, this study is motivated to explore the purchase intention of silver consumers in SFV platforms. We categorize the characteristics of SFV platforms into social and technical aspects, and analyze silver consumers’ purchasing decisions in terms of existence, relatedness, and growth needs. The empirical results of 284 samples show that social belonging, perceived trust, and product relevance are positive factors that promote purchase. Information diversity and social interaction have significant positive effects on social belonging and perceived trust. Recommendation affordance is significantly positively associated with perceived trust and product relevance, while platform ease of use did not have a significant effect on perceived trust. The findings provide management insights into SFV platforms to better understand the digital divide faced by silver consumers and to facilitate increased consumption.

## 1. Introduction

Embedding advertisements (ads) in short-form video (SFV) sequences to convert scarce attention resources into purchasing behavior has become a new business model to reach customers and promote products [1]. Unlike traditional e-commerce, SFV e-commerce no longer needs to accumulate audience resources for a long time, but through the industry chain and traffic redirecting to achieve rapid traffic monetization [2]. Meanwhile, “silver consumers” (consumers over the age of 50) make up a growing portion of society’s demographics, and the group’s extensive use of SFV apps has stimulated demand for differentiated consumption within the platform. While digital technology is driving the SFV and consumer industries into a new stage of development, it also brings new challenges for silver consumers, such as the digital divide, digital payments, and network security issues [3,4]. These challenges mean that despite all potential advantages of digital technology, the elderly are less likely to have access to and to exploit the potential of internet usage and short-form video platforms in general [5]. The research objective of this paper is to reveal the unique purchase decision mechanism of silver consumers in SFV platforms, i.e., how the sociotechnical characteristics of platforms promote silver consumers’ purchase intentions by influencing their existence, relatedness, and growth (ERG) needs.

With the aging of the population and the popularization of digital technology, the proportion of the silver group with money and leisure to use SFV e-commerce will increase accordingly. SFV companies should pay more attention to the size and potential of the silver market, and help silver groups to cross the digital divide and remove barriers to the use of SFV e-commerce platforms. In terms of research on silver consumers and digital technology, there is thus a need for more research investigating how digital platforms can assist silver consumers (e.g., increase ability and reduce vulnerability) to respond to marketing changes brought about by SFV technologies. However, few studies [6,7,8,9] on SFV e-commerce have focused on the decision-making mechanism and behavioral characteristics of silver consumers. Previous purchase intention models [6,7,8,9] in social e-commerce are not directly applicable to silver consumers, as there are significant differences in marketing perceptions and information behaviors between the elderly and young and middle-aged groups [10].

Due to the lack of objective conditions and subjective integration, the silver group are becoming awkward characters at the margins of the digital age. From the perspective of objective interests, the e-commerce market usually focuses on young and middle-aged people who are skilled in the use of digital technology, but neglects to tilt its attention toward the elderly [11]. In terms of individual characteristics, the silver group lacks the necessary digital and media literacy, and their ability and willingness to use digital media are lower. Because of aging, the silver group faces specific limitations in processing and understanding information, which puts them at a disadvantage in commercial transactions. The ability to comprehend and process marketing information declines with aging, largely because of the taxing mental effort required for the elderly to engage in cognitive processes of product interpretation [12]. Silver consumers are more likely to form purchase judgments by processing small amounts of subjective and emotional information rather than objective information that may be processed through a central (cognitive) pathway. In conclusion, the online consumption behavior of silver consumers shows uniqueness, and they may need stronger group emotional support and seek the security and trust of the purchasing environment to compensate for the lack of cognitive ability.

Therefore, this study focuses on SFV e-commerce platforms and tries to reveal the purchase decision mechanism of silver consumers from the perspective of ERG needs, thus providing managerial and practical insights into activating the potential of the senior market. The ERG theory, which is primarily used to analyze the drivers of human behavior, emphasizes the integration of an individual’s needs into three dimensions: existence (e.g., functional needs), relatedness (e.g., belonging, togetherness), and growth (e.g., self-actualization, self-esteem). Given that the success of SFV platforms derives from a combination of social and technical systems, we first categorize the key characteristics of SFV platforms into social and technical aspects based on the sociotechnical systems theory and previous literature. Then, satisfying user needs is the primary goal of information system design, and the information behavior of silver consumers in SFV platforms originates from the corresponding needs. We therefore analyze the behavioral motivation of silver consumers’ purchasing decision based on the ERG theory [13] from three aspects: existence needs, relatedness needs, and growth needs. Specifically, a survey is implemented to explore how the social and technical characteristics of SFV e-commerce systems influence the ERG needs of silver consumers, thereby facilitating purchase intentions.

Based on the social and technical characteristics of the SFV platform system, we analyze the cognitive process of silver consumers’ purchase decision by considering their psychological perception and environmental interaction comprehensively. This study is an early exploration of the purchasing mechanisms of silver consumers in SFV social platforms, and makes theoretical contributions to the research field related to the sustainable digital well-being of silver consumers.

## 2. Theory Background and Hypothesis Development

### 2.1. SFV-Driven E-Commerce

SFV-driven e-commerce refers to a business model that carries out e-commerce activities through content creation and online marketing on SFV platforms. In recent years, SFV apps represented by TikTok and Douyin have swept the world, and the “SFV plus e-commerce” model has gradually been recognized by developers and consumers. Compared with traditional e-commerce, the display mode combining SFV, live streaming, and mall in SFV platform can deliver real-time multidimensional information. In this case, consumers can understand the product attributes through a variety of ways, and will be stimulated by richer content and decision-making guidance. In addition, the interactivity of SFV e-commerce is no longer limited to reviews and customer service, as multimodal video communication empowers consumers with immersive experiences [14]. Real-time bullet chatting and virtualized interactive props in live streaming can enhance social interaction, thus alleviating the barriers to live streaming for silver consumers and enhancing their product perception. According to the modality effect [15], video explanations are easier to understand and have a lower learning load than purely graphic learning. At the level of human–computer interaction, SFV e-commerce also has the advantage of ease of use [16], and the perceived trust of online shoppers relies to some extent on perceived ease of use. In addition to content and interaction perspectives, personalized recommendation technologies of SFV platforms play an obvious role in driving user stickiness and consumption behaviors [17]. Recommender systems not only provide users with opportunities to take actions, but also endow platforms with core competencies driven by both content and technology. At present, the social, advertising and e-commerce components of many SFV platforms are centering their business layout around the attention resources brought about by accurate personalized recommendations.

The SFV platform is a typical composite system of social and technical systems, requiring both social capitals to support community operations and information technology for system design and content services. The social and technical systems in the platform are interdependent and influence each other. Given that the sociotechnical approach is a general framework for analyzing the usefulness of a system from the perspective of social and technical factors, we categorize the key characteristics of SFV e-commerce platforms into social and technical aspects based on sociotechnical systems theory and previous literature. The social aspect refers to the role of the platform in maintaining social interactions and facilitating community exchanges, including information diversity and social interaction. The technical aspect focuses on human–computer interaction and predicting user preferences through artificial intelligence, including ease of use and recommendation affordance.

Current research on SFV e-commerce focuses on the factors that positively influence users’ willingness to buy in SFV scenarios, including the addiction mechanism of APP use [6], content quality, relationship quality [7], entertainment [8], interaction based on live streaming technology, and intelligent recommender systems [9]. These factors help us understand how SFV advertising affects consumer engagement behavior and explain why SFV e-commerce has become a new marketing channel on a global scale. However, this literature has not focused on the potential impact of silver consumers, despite the increasing activity of the elderly population on SFV platforms, and even many elderly users heavily rely on SFV platforms for information gathering, online entertainment, and social interaction. Therefore, this paper will focus on how silver consumers fulfill various needs in the SFV platform and help them enjoy better e-commerce services by revealing the unique interaction mechanisms of silver consumers.

### 2.2. The ERG Needs of Silver Consumers

In online networking, providing silver groups with tailored messages, feedback, and goals that meet their specific needs is critical to triggering behavior change. Therefore, we analyze the needs of silver consumers based on the ERG theory [13] from three aspects: existence needs, relatedness needs, and growth needs, which can trigger potential purchase intention. The ERG theory, which is primarily used to analyze the drivers of human behavior, integrates the needs of individuals into three levels: existence (e.g., functional needs), relatedness (e.g., belonging, togetherness), and growth (e.g., self-actualization, self-esteem). In this paper, we extend the ERG theory to online interaction scenarios, and combine the social and technical attributes of SFV platforms to understand and infer the ERG needs of silver consumers.

First, in short video platforms, the existence needs of silver consumers are no longer only related to material desires, but also how to form trust in the platforms through familiarity with software use and information acquisition. Once an individual is unable to build platform trust, he or she will not continue to “exist” on the platform, i.e., will give up using the platform. Silver consumers pay more attention to safety and trust in online shopping, and have higher requirements for after-sales service. In eHealth service scenarios, perceived trust was found to be a major factor in service adoption among the silver group [18]. In a study of travel app adoption, it was found that the silver group’s technology trust is formed based on user experience, and that technology trust has a significant effect on the silver group’s adoption willingness [19].

Second, the relatedness needs of silver consumers in the SFV platform are reflected in their desire for social belonging. Elderly people often lack family companionship, and thus have a stronger need to seek emotional support from a community [20]. Some silver groups turn to consumer communities to form their own social relationships, thus enhancing their sense of social belonging [21,22]. Social isolation and emotional loneliness drive the silver group to seek social experiences, and interactions with salespeople can increase silver consumers’ willingness to buy [23]. In addition, there is a group purchasing effect among silver consumers, who are willing to exchange product information and tend to consult their peers before purchasing. When the herd behavior theory is used to study online shopping decisions, the group effect was found to have an important influence on the purchasing behavior of silver consumers [24].

Third, silver consumers’ growth needs in SFV platforms are more about discovering interesting content and increasing opportunities for more action, which need to be supported by recommender systems. The growth and development needs of the platform users belong to a self-productive effect such as the ability to seek knowledge, to build psychological connection, to develop personality. Silver consumers have relatively conservative behavioral patterns on e-commerce platforms, preferring a simple purchase process and relying more on advertisements and recommendations when obtaining information about products and services. Research on the psychological sense of brand community suggests that advertising may be effective in creating a psychological connection with silver consumers and promoting brand consumption [25]. As the core guarantee that supports the content-technology dual-channel operation of the SFV platform, the recommender system is the implicit driver that prompts users to have a creative or productive impact on themselves and the interactive environments. User perceptions of algorithmic availability help to enhance the user growth experience and promote co-evolution of users and algorithms (i.e., a positive feedback loop between continued use and algorithmic efficiency) [26]. Therefore, the long-term growth needs of silver consumers on SFV platforms can be reflected as the relevance of recommended information, i.e., product relevance. Higher product relevance helps silver consumers efficiently and quickly acquire product information, build a preference for personalized products, and thus generate a more favorable advertising attitude.

### 2.3. The Sociotechnical Characteristics of SFV Platforms

#### 2.3.1. Information Diversity

Information diversity includes informativeness and variety. Informativeness is the amount of information embedded in seller-created and buyer-created content [27]. Diversity refers to the diversity of information dissemination media (e.g., short videos, live streaming, video comments, live pop-ups) and the diversity of information content. Information diversity has been shown to have a direct or moderating effect on e-commerce product sales [28].

According to emotional contagion theory [29], the diverse and interesting informational content of short videos is more likely to evoke emotional arousal in consumers. Emotion, as one of the most significant reasons for stimulating consumer behavior, can enhance consumers’ social presence and willingness to purchase [30]. In social mediums, by sharing highly personal content that revolves around their lifestyle and interests, users can forge deeper psychological bonds with their partners, which in turn enhances a sense of social belonging [31].

The diversity of information forms and content forms can increase users’ involvement in the information of products. Involvement theory refers to the different degrees of attention to things after being stimulated, including situation involvement, enduring involvement, and response involvement [32]. Depending on how the individual behaves when dealing with the involved object, the involvement zone can be categorized into product involvement, advertising involvement, purchase decision involvement, and consumption involvement [33,34]. Silver consumers are more rational than other groups when shopping and have higher demands for information diversity. Traditional e-commerce platforms are more single in information presentation than SFV platforms, which cannot consider above four kinds of involvement, so silver consumers are relatively more involved in SFV platforms. A higher level of involvement means a higher level of knowledge about the product, the merchant, and a growth in trust. Therefore, we hypothesize as follows:

**H1a.**  *The information diversity of SFVs positively affects the social belonging of silver consumers.*

**H1b.**  *The information diversity of SFVs positively affects the perceived trust of silver consumers.*

#### 2.3.2. Social Interaction

In social commerce, new technological features such as user stickiness, personalization, and virtual socialization can facilitate collaboration between users and businesses, and between users and users [35,36]. Some platforms support efficient social interaction or information exchange through video presentations, 3D displays, hyperlinks, etc. [37,38].

The social interaction of SFVs is reflected in two aspects, one is the interaction between users and anchors or other users on the platform. According to the interaction ritual chains theory, joint participation in an emotionally driven symbolic event can result in the creation or enhancement of collective identity and emotional energy [39], which further enhances willingness, trust, and loyalty for continued interaction [40].

Another aspect of the social interaction of SFVs is that users utilize the platform as a medium to interact with their friends and relatives. SFV communities can alleviate the weakening of the social network of silver groups in real life, and reduce the sense of loneliness and satisfy the need for sociality, thus enhancing social participation and sense of social belonging [41]. In addition, previous research on the impact of advert effectiveness has shown that social interaction is an important factor in enhancing the effectiveness of platform adverts. The more interactive an online advert is, the more likely it is to elicit user feedback and positive perceptions, thus increasing perceived trust [42]. Accordingly, we propose the following hypotheses to test whether the social interaction of SFV platforms still has a similar impact on silver consumers:

**H2a.**  *The social interaction of SFVs positively affects the social belonging of silver consumers.*

**H2b.**  *The social interaction of SFVs positively affects the perceived trust of silver consumers.*

#### 2.3.3. Ease of Use

The convenience of information will promote customer consumption [43]. Perceived ease of use and channel ease of use are prerequisites for generating consumption behavior and key factors influencing consumption decisions [44]. In general, product information is the easiest and earliest product-related content for consumers to access. The more readily available the product information, the easier it is for the consumer to accept the product. When information is difficult to obtain, consumers are less informed about products and perceived risk grows, leading to less consumption. In SFV e-commerce, a simple swipe up provides access to a wealth of information. The availability of product information, as explained by a sales anchor, may enhance perceived trust [35]. In addition, early research has shown that perceived ease of use affects not only the initial adoption of information systems by users, but also the willingness to continue to use [45].

However, whether this sustainable impact is significant among silver consumers needs to be further explored, because software operation involvement does not mean that silver consumers with lower information processing skills can generate subsequent product involvement, advertising involvement, purchase decision involvement, and consumption involvement. Silver consumers generally have fear of unfamiliar and novel things and fear of loss, which represses a large part of consumption demand. The breakthrough of psychological barriers for silver consumers and the establishment of product trust may be more influenced by video emotion, content interest, and social interaction. The following hypothesis is thus proposed to test whether the perceived trust of silver consumers in SFV scenarios is affected by ease of use:

**H3.**  *The ease of use of SFVs positively affects the perceived trust of silver consumers.*

#### 2.3.4. Recommendation Affordance

“Affordance” is first proposed by the field of ecopsychology and refers to the “harmonization of the environment with the animal.” Norman [46] introduces affordance into design science to explore the relationship between human perception and technology or objects, and then the affordance theory is widely studied in human–computer interaction. Schrock [47] argues that affordance is the interplay between objective attributes of technology and subjective perceptions of utility, and interprets information behavior in social media through the lens of affordance.

Recommendation affordance refers to the possibility of SFV platforms to provide users with personalized information and services through user behavior and intelligent recommendation algorithms [48]. Recommendation algorithms promote product relevance, and recommendation affordance influences the user’s perception of product relevance by shaping and forming the interaction environment [49]. Meanwhile, the recommender system, through the optimization strategy and the distribution of high-quality content, enables high-quality products to reap more exposure, which to a certain extent can positively affect the perceived trust of silver consumers [26]. If SFV platforms can consistently provide accurate recommendations, the needs of silver consumers will be satisfied, thus gradually generating a sense of trust and dependence. Therefore, we postulate that:

**H4a.**  *The recommendation affordances of SFVs positively affects the product relevance.*

**H4b.**  *The recommendation affordances of SFVs positively affects the perceived trust of silver consumers.*

### 2.4. Purchasing Intentions of Silver Consumers

#### 2.4.1. The Influence of Social Belonging

Social belonging is defined as “the sense that one is a part of a readily available, mutually supportive network of relationship” and consists of four components: membership, mutual influence, need fulfillment, and shared emotions [50]. When silver consumers actively participate in social groups, it not only gains self-esteem and happiness, but also a sense of belonging. The fan economy states that fans create an emotional connection through discussion and sharing, which in turn influences the choice of products [51]. Similarly, social belonging can stimulate empathy and sympathy in the minds of silver consumers, which is a deep-seated psychology that is likely to have a direct impact on purchasing decisions. In SFV virtual communities, silver consumers can seek more emotional support, group identity, and social belonging. These emotional supports can increase consumers’ brand recognition [52] and promote continuous consumption and word-of-mouth communication [53]. Hence, we propose:

**H5.**  *Silver consumers’ social belonging positively affects purchase intention.*

#### 2.4.2. The Influence of Perceived Trust

Trust is a key factor in enabling online transactions. The quality of information and security measures on platforms can affect consumers’ perceived trust, which can potentially influence purchases [54]. Perceived trust can be analyzed from two perspectives: the platform and the seller. Perceived trust in the platform refers to the consumers’ perception of the institutional structure of the platform, while perceived trust in the seller is the feeling of the seller’s product presentation [55]. Basically, silver consumers have a higher need for trust in the platform and the interaction object, because of their psychological characteristics and factors such as barriers to Internet use. In e-health scenarios, perceived trust plays a key role in service acceptance among the elderly, and users’ technological anxiety will reinforce the role of affective trust [56]. The following hypothesis is thus proposed to test the role of perceived trust in SFV e-commerce platforms for silver consumers:

**H6.**  *Silver consumers’ perceived trust positively affects purchase intention.*

#### 2.4.3. The Influence of Product Relevance

According to the self-reference effect [57], individuals are more likely to be persuaded by self-relevant information. For example, relevance is an important indicator for evaluating the effectiveness of adverts [58], and higher relevance significantly reduces consumers’ advert avoidance behavior [59]. Personal relevance is a perceived connection between an individual’s needs, goals, values, and product information [60]. In this psychological evaluation process, consumers assess the extent to which a product message is self-relevant or the extent to which it satisfies their needs, goals, and values. When many international brands enter a local market, they often pick a local spokesperson to promote the relevance of the product to local potential consumers. In SFV marketing, we emulate advert relevance and personal relevance by defining product relevance as the consumer’s perception of the utility and usefulness of a targeted advert at the cognitive, affective, and behavioral belief level. The perceived relevance of the product in the recommended video promotes positive consumer perceptions, emotions, and behaviors that increase purchase persuasion. Considering the above-mentioned arguments, we posit:

**H7.**  *Product relevance positively affects the purchase intention of silver consumers.*

**Figure 1:**
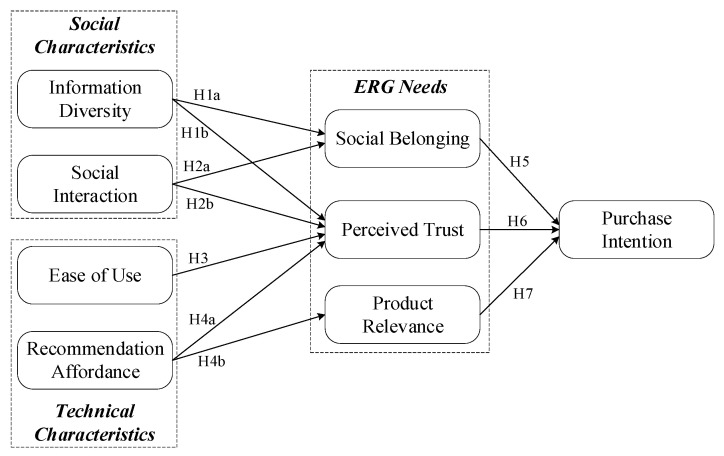
The conceptual model.

The overall conceptual model for this study is shown in Figure 1. We categorize SFV platform characteristics based on sociotechnical systems theory and use silver consumers’ existence, relatedness, and growth needs as mediators of the influence of platform characteristics on purchase intention.

## 3. Survey Design

The survey includes eight constructs, which are measured from previous studies. Some of the questions are adapted to fit the context and hypotheses of this study. All questions use a 5-point Likert scale from “disagree” (1) to “agree” (5) (see Appendix A for details).

### 3.1. Pre-Survey

In this study, silver groups with experience of using SFV platforms were selected as survey respondents, and a total of 68 samples were collected for pre-survey. The purpose of the pre-survey was to test the reasonableness of our questionnaire design with a small sample size and to further improve the questionnaire content and questioning style in response to respondents’ feedback. To test the reliability of the questionnaire, we examined the Cronbach alpha coefficients of the eight constructs and the corrected item-total correlation (CITC). The test results showed that the CITC values [61] for all constructs were greater than 0.3 and the Cronbach alpha coefficients [62] were greater than 0.8, indicating the reliable reliability of the scale. The scales were based on previous relevant studies, so the reasonableness of the factor analysis was first ensured by the KMO test and Bartlett’s spherical test, and then the structural validity of the scale was tested by using principal component analysis and maximum variance orthogonal rotation. The test results showed that the KMO values of the eight constructs are all greater than 0.8 [63], which is suitable for factor analysis. The results of the factor analysis showed that the dimensions obtained from the factor analysis of the items were basically consistent with the previous design. The factor loadings of each item on their respective dimensions were all greater than 0.6 [64], with good structural validity.

### 3.2. Data Collection

This study was conducted for the silver group over 50 years old, and questionnaire links were distributed through social software such as QQ (v8.9.69.603) and WeChat (v8.0.42), as well as SFV platforms such as Douyin (v24.1.0) and Kuaishou (v11.11.10.34366). The questionnaire was filled out through the leading online survey platform in China (https://www.wjx.cn, accessed on 1 February 2023), which has been used for data collection in many academic studies [65,66]. Combined with offline questionnaire distribution, we collected 370 questionnaires. Excluding responses that were too short to fill out and those with no online shopping experience, we ended up with 284 valid questionnaires. The descriptive statistical information of the questionnaires is shown in Table 1.

The total number of samples was 284, of which 97 were males, accounting for 34.15%. In terms of age, the silver group aged 50–55 accounted for 52.82% of the total sample, 56–60 accounted for 34.51%, and 61–65 accounted for 7.75%, which is roughly in line with China’s current age distribution of the elderly.

## 4. Analysis and Results

Based on SmartPLS 3.0, the research model was analyzed and tested using Partial Least Squares (PLS) method. We chose the PLS method for two reasons. First, PLS is well suited for testing complex relationships and can avoid infeasible scenarios and factor uncertainty, so it is suitable for developing exploratory research theories [67]. Second, PLS is not limited to normally distributed samples, while being able to model under conditions of small sample size [68] and focusing on maximizing the explained variance of endogenous variables [69].

### 4.1. Reliability and Validity Analysis

Cronbach’s alpha (CA) was adopted to test the reliability of the questionnaires. Composite reliability (C.R.) and average variance extracted (AVE) were employed to test the convergent validity. As shown in Table 2, the Cronbach alpha coefficients and composite reliability for all constructs in this study are greater than the threshold of 0.7, and the AVE values are greater than the threshold of 0.5 [70], which suggests the acceptable reliability and convergent validity.

We then performed validity checks. First, the square root of the AVE for each construct was greater than its correlations with the other constructs (Table 2), indicating that the model has sufficient discriminant validity [70]. Second, the item loadings of each construct were significantly higher than the cross-loadings on the other constructs (Table 3), indicating that the measurement model in this study has good discriminant validity [70]. Third, heterotrait–monotrait ratio (HTMT) is commonly used to assess the discriminant validity of measurement models [71]. As shown in Table 4, all HTMT values were below 0.85, indicating that the measurement model has good discriminant validity [72]. In summary, the measurement model in this study has good reliability, convergent validity, and discriminant validity for structural equation modeling.

### 4.2. Structural Model

The bootstrapping algorithm was executed based on 5000 resamplings to verify the research hypotheses (Table 5). The quality of the structural model was assessed by the R-square of the endogenous variables, the Q-square of the Stone–Geisser test [73], and the significance of the path coefficients. The R-squared exceeds 0.20, indicating that the model has good explanatory power. In addition, we calculated the Q-squared value of the Stone–Geisser test as a criterion for predicting correlation. The results of the blindfolding algorithm showed that the Q square values of SB, PT, PR, and PI are 0.196, 0.220, 0.148, and 0.327, respectively, which are all greater than zero, indicating that the PLS path model has prediction accuracy and correlation.

### 4.3. Results

We first analyzed the effects of information diversity, social interaction, ease of use, and recommendation affordance on social belonging, perceived trust, and product relevance in SFV platforms. The results showed that information diversity (β = 0.149, p < 0.01) and social interaction (β = 0.471, p < 0.001) had a significant positive effect on social belonging, and the R-squared value indicated that information diversity and social interaction together explained 28.9% of the variance in social belonging. Meanwhile, information diversity (β = 0.220, p < 0.001), social interaction (β = 0.188, p < 0.01) and recommendation affordance (β = 0.245, p < 0.01) had a significant positive effect on perceived trust, while ease of use did not have a significant effect on perceived trust. The R-squared value indicates that information diversity, social interaction, and recommendation affordance together explain 31.7% of the variance in perceived trust. In addition, recommendation affordance (β = 0.448, p < 0.001) had a significant positive effect on product relevance and explained 20.1% of the variance in product relevance.

At the final behavioral decision level, we verified the effects of social belonging, perceived trust, and product relevance on purchase intention. The results showed that the positive effects of social belonging (β = 0.221, p < 0.001), perceived trust (β = 0.220, p < 0.001), and product relevance (β = 0.391, p < 0.001) on purchase intention were significant, and R-squared values indicated that these three variables explain 46.0% of the variance in purchase intention. In summary, the above results support all hypotheses except H3.

We also implemented a mediation effects analysis for the model. The results in Table 6 report the indirect effects and the variance accounted for (VAF) [74]. VAF represents the size of the indirect effect relative to the total effect. As can be seen, the findings reveal how the six mediating paths (except for “EU path”) are positive and significant.

## 5. Discussion and Conclusions

### 5.1. Results Discussion

This study takes the silver group in SFV platforms as the research object, and proposes a purchase decision model of silver consumers based on sociotechnical systems theory and the ERG theory. The empirical analysis conclusions of this study are summarized as follows.

First, in terms of the characteristics of SFV platforms, information diversity and social interaction have a significant positive effect on the social belonging. Information diversity, social interaction, and recommendation affordance can promote perceived trust in the silver group, while recommendation affordance is positively related to consumers’ perceived product relevance. Unexpectedly, ease of use does not have a significant effect on perceived trust, probably because the establishment of trust among silver consumers is more influenced by the content emotion, content appeal, and social interaction of SFVs. Further, the ease of use of SFV platform operation does not mean that it will improve silver consumers’ product involvement.

Second, we analyze based on the characteristics of silver consumers. Social belonging, perceived trust, and product relevance can positively influence the purchase intention of silver consumers. Social belonging and perceived trust bring group emotional support to silver consumers, which plays an important role in their purchasing behavior. Product relevance can trigger the passive needs that consumers themselves were not previously aware of, and easily make silver consumers think that the product is self-relevant, thus promoting the positive consumption behavior.

Third, the silver group on SFV platforms has the following characteristics: the elderly aged 50–55 account for a high proportion of the users and consumers, and higher age groups do not have a high acceptance of SFV e-commerce. Many silver groups have been using SFVs for a long time, mainly focusing on the mainstream platforms of Douyin, Kuaishou and WeChat. The purchase rate of the silver group in SFV e-commerce is not high, so there is still a large scale and possibility of the silver market for SFV companies.

To summarize, the silver economy is likely to be a market segment that holds great potential in the future, as the trend of SFV e-commerce rising in the silver group is obvious. However, the SFV industry has not yet formed the corresponding content support, and there is still a lot of room to play in the elderly information behavior assistance and segmented category operation. SFV e-commerce enterprises should take the initiative to meet the needs of the silver group, which is trending towards diversification, personalization, and intelligence, and clear the obstacles for the silver group to enjoy better digital dividends.

### 5.2. Theoretical Contributions

As the affluent baby-boomer segment rapidly approaches retirement, marketers are becoming more aware of elderly consumers. In recent years, SFV apps have become popular among young groups around the world, and embedding e-commerce components in SFVs has become an emerging means of attention marketing. However, the social and technical system of SFV platforms also attracts a group of older adults with needs for consumption, information sharing, and emotional companionship. Issues such as whether the silver group can enjoy the digital marketing dividend of SFV industry and whether digital payment is friendly to the silver group have naturally become unavoidable social, ethical, and commercial issues.

This study is an early exploration of the mechanisms of silver consumers’ purchasing behavior in SFV e-commerce platforms. We reveal the consumption psychology and behavioral logic of silver consumers, and make a theoretical contribution to the research field related to the sustainable digital well-being of silver consumers. In addition, we find that the information diversity, social interaction, and recommendation affordance of SFV platforms are key factors influencing the purchase decisions of silver consumers. This implies that the social and technical systems of are equally important to silver consumers. Platforms need both social capitals to maintain user bonds and information technology for content distribution.

### 5.3. Managerial Implications

The digital economy continues to impact the silver group, and the potential dividends brought by silver consumers in the e-commerce consumption structure cannot be ignored. SFV companies urgently need to understand the intrinsic needs and behavioral characteristics of consumers in this market segment, and then leverage the social and technological features of the platform system to support the inclusion of silver consumers. This study provides the following management insights into SFV e-commerce operations targeting silver consumers.

(1) Differentiated precision operation

SFV e-commerce managers should realize that the psychological characteristics of the silver group are special. Silver consumers have a more conservative consumption concept, so perceived trust helps them integrate into the virtual community. According to the findings of this study, perceived trust may be more influenced by community interaction and content quality. Therefore, platforms can rely on professional teams to create “silver influencers” to realize user retention and SFV marketing through the natural perceived trust among the elderly. For example, the platform can popularize health knowledge through the official accounts of major hospitals and doctors to expand its influence on the silver community. In addition, the platform can leverage intelligent algorithms to improve content distribution efficiency and capture the differentiated needs of the silver community.

(2) Expanding the monetization space

Internet traffic monetization refers to the conversion of Internet traffic into cash through certain means. At present, SFV platforms mainly rely on advertising, e-commerce, live streaming, knowledge payment, publishing, and other channels for traffic monetization. The silver group’s traffic monetization space may be compressed as they struggle to generate sustained interest in purchase channels that require more cognitive effort to be invested. Thus, SFV platforms can take measures to create more consumption opportunities for the silver group. For example, the platform can support the well-known elderly celebrity, through their strong influence on advertising placement and dissemination, to create a natural community shopping atmosphere. By negotiating with suppliers, the platform can provide high-quality and affordable fishing gear, kitchenware, clothing and other products suitable for silver consumers. In the payment knowledge sharing community, platforms can embed relevant courses for the silver group and provide them with high-quality information resources.

### 5.4. Conclusions

The increasing rise of SFV platforms has not only become an important entertainment for people to spend their fragmented time on, but also a new marketing and sales channel for enterprises. Given the consumption potential of the silver group in SFV e-commerce and the barriers they face in the use of digital technology, this paper empirically investigates the purchase decisions of silver consumers in SFV platforms based on sociotechnical systems theory and the ERG theory. The results found that social belonging, perceived trust, and product relevance are positive factors that promote the purchase decisions of silver consumers. Information diversity and social interaction help silver consumers to improve social belonging and perceived trust. Recommendation affordance is significantly positively associated with perceived trust and product relevance, while ease of use has no significant effect on perceived trust. We extend the application of the ERG theory to the SFV platform and empirically demonstrate that the platform’s social characteristics and intelligent recommender system can enhance the existence, relatedness, and growth needs of silver consumers. This study helps SFV platforms to comprehensively understand the digital divide faced by silver consumers and promotes increased consumption.

## 6. Limitations and Future Research

Three limitations exist in our study from which future avenues of research could spawn. First, the target population of this study is the silver group who have experience in SFV shopping or have the behavior of using short videos, but those who do not have the above behaviors are also potential customers. The reliability of our model can be further validated by investigating these potential customers in future studies. Second, we did not break down the silver hair group further, e.g., full-time silvers (i.e., people who remain on the labor market), retired silvers (i.e., enjoying the charms of more free time and still relatively good health), and seniors over 80 years of age (i.e., people with increasing health problems). The characteristics of different types of silver groups will largely determine their habits, routine and purchasing decisions. Future research could further segment the silver group to reveal silver consumer behavior from multiple perspectives. Third, some inappropriate design or functionality flaws of the SFV platform (e.g., vulgar content) may negatively affect the purchase decisions of silver consumers, and this is an aspect that needs to be considered in future work.

## Figures and Tables

**Table 1:** Descriptive statistics of respondents.

Demographics		Frequency	Percentage
Gender	Male	97	34.15%
	Female	187	65.85%
Age	50–55	150	52.82%
	56–60	98	34.51%
	61–65	22	7.75%
	66 or older	14	4.93%
Frequency of SFV platforms using (minutes per day)	<30	113	39.79%
	30–60	101	35.56%
	>60	70	24.65%
Frequency of SFV shopping	Never	70	24.65%
	Occasionally	142	50.00%
	Often	72	25.35%
Short video platforms used (multiple choice)	Douyin	183	64.44%
	Kuaishou	144	50.70%
	WeChat Video	139	48.94%
	Xigua Video	74	26.06%
	Xiaohongshu	28	9.86%
	Others	19	6.69%

**Table 2:** Reliability and validity testing.

Construct	CA	C.R.	AVE	ID	SI	EU	RA	SB	PT	PR	PI
ID	0.841	0.894	0.678	**0.804**							
SI	0.861	0.905	0.706	0.319	**0.840**						
EU	0.824	0.879	0.646	0.345	0.392	**0.823**					
RA	0.853	0.901	0.694	0.368	0.344	0.384	**0.833**				
SB	0.860	0.905	0.704	0.299	0.519	0.431	0.459	**0.839**			
PT	0.871	0.912	0.721	0.413	0.391	0.367	0.438	0.531	**0.849**		
PR	0.901	0.931	0.772	0.488	0.459	0.534	0.448	0.469	0.512	**0.878**	
PI	0.859	0.904	0.703	0.495	0.502	0.481	0.534	0.511	0.532	0.602	**0.838**

Notes: Diagonal elements in bold represent the square root of the average variance extracted (AVE).

**Table 3:** Item loadings and cross loadings.

Construct	Indicator	ID	SI	EU	RA	SB	PT	PR	PI
ID	ID1	**0.724**	0.109	0.174	0.244	0.073	0.200	0.236	0.288
	ID2	**0.803**	0.342	0.336	0.295	0.266	0.306	0.376	0.337
	ID3	**0.876**	0.314	0.318	0.366	0.314	0.420	0.490	0.504
	ID4	**0.805**	0.187	0.237	0.252	0.220	0.332	0.389	0.405
SI	SI1	0.285	**0.842**	0.302	0.302	0.424	0.350	0.361	0.398
	SI2	0.294	**0.864**	0.386	0.280	0.487	0.337	0.464	0.437
	SI3	0.252	**0.814**	0.312	0.314	0.425	0.352	0.340	0.456
	SI4	0.234	**0.838**	0.311	0.258	0.398	0.265	0.370	0.391
EU	EU1	0.272	0.303	**0.820**	0.324	0.400	0.274	0.402	0.412
	EU2	0.358	0.391	**0.845**	0.267	0.371	0.294	0.550	0.430
	EU3	0.298	0.348	**0.857**	0.361	0.375	0.348	0.477	0.393
	EU4	0.205	0.243	**0.769**	0.307	0.273	0.283	0.318	0.353
RA	RA1	0.317	0.219	0.270	**0.789**	0.290	0.333	0.316	0.460
	RA2	0.318	0.380	0.340	**0.832**	0.438	0.418	0.423	0.478
	RA3	0.357	0.267	0.341	**0.849**	0.409	0.363	0.367	0.429
	RA4	0.233	0.258	0.321	**0.860**	0.374	0.335	0.375	0.408
SB	SB1	0.247	0.452	0.421	0.401	**0.838**	0.435	0.446	0.457
	SB2	0.268	0.448	0.389	0.378	**0.867**	0.430	0.416	0.410
	SB3	0.287	0.496	0.373	0.465	**0.874**	0.523	0.420	0.464
	SB4	0.191	0.320	0.241	0.269	**0.772**	0.380	0.268	0.373
PT	PT1	0.348	0.321	0.358	0.372	0.477	**0.842**	0.461	0.463
	PT2	0.365	0.367	0.321	0.382	0.390	**0.842**	0.425	0.427
	PT3	0.377	0.367	0.293	0.382	0.507	**0.863**	0.419	0.452
	PT4	0.309	0.268	0.271	0.350	0.426	**0.849**	0.435	0.465
PR	PR1	0.428	0.395	0.434	0.385	0.418	0.468	**0.871**	0.540
	PR2	0.443	0.459	0.520	0.435	0.434	0.438	**0.896**	0.556
	PR3	0.478	0.402	0.473	0.373	0.411	0.480	**0.869**	0.500
	PR4	0.367	0.352	0.445	0.379	0.384	0.417	**0.878**	0.518
PI	PI1	0.423	0.405	0.401	0.420	0.412	0.425	0.472	**0.842**
	PI2	0.382	0.431	0.373	0.469	0.407	0.452	0.468	**0.815**
	PI3	0.476	0.452	0.448	0.452	0.478	0.500	0.593	**0.883**
	PI4	0.371	0.392	0.385	0.453	0.410	0.398	0.474	**0.813**

Notes: The loadings on principal factors are in bold and all higher than 0.7.

**Table 4:** Heterotrait–monotrait ratio (HTMT).

	1	2	3	4	5	6	7
(1) ID							
(2) SI	0.348						
(3) EU	0.395	0.456					
(4) RA	0.426	0.393	0.449				
(5) SB	0.318	0.592	0.499	0.520			
(6) PT	0.458	0.447	0.425	0.504	0.608		
(7) PR	0.535	0.517	0.608	0.505	0.524	0.579	
(8) PI	0.560	0.581	0.565	0.623	0.589	0.612	0.679

**Table 5:** Hypotheses summary.

Hypothesis	β	T Statistics	*p*-Value	Hypothesis Testing
H1a: ID → SB	0.149 **	2.690	0.007	Supported
H1b: ID → PT	0.220 ***	3.668	0.000	Supported
H2a: SI → SB	0.471 ***	8.323	0.000	Supported
H2b: SI → PT	0.188 **	2.741	0.006	Supported
H3: EU → PT	0.123	1.942	0.052	Not supported
H4a: RA → PT	0.245 ***	4.000	0.000	Supported
H4b: RA → PR	0.448 ***	7.631	0.000	Supported
H5: SB → PI	0.211 ***	3.292	0.001	Supported
H6: PT → PI	0.220 ***	3.476	0.001	Supported
H7: PR → PI	0.391 ***	6.076	0.000	Supported

Note: ** *p* < 0.01, *** *p* < 0.001.

**Table 6:** Path coefficients of mediation analysis in PLS-SEM.

Path	Original Sample	Sample Mean	Standard Deviation	T Statistics	VAF
SI → PT → PI	0.043	0.043	0.021	2.085 *	31.4%
RA → PR → PI	0.172	0.174	0.042	4.093 ***	75.4%
RA → PT → PI	0.056	0.057	0.022	2.516 *	24.6%
ID → PT → PI	0.051	0.052	0.021	2.448 *	63.8%
ID → SB → PI	0.03	0.032	0.016	1.869 *	37.5%
SI → SB → PI	0.094	0.095	0.033	2.864 ***	68.6%
EU → PT → PI	0.028	0.029	0.017	1.656	/

Note: * *p* < 0.1, *** *p* < 0.001.

## Data Availability

Data will be made available on request.

## Appendix A

**Table A1:** Questionnaire items.

Construct	Item	Reference
Information Diversity (ID)	ID1. I watch video presentations as well as buyer reviews and pictures ID2. I can get information on SFV platforms that I cannot get on other platforms. ID3. I think video presentations provide a more comprehensive view of the products. ID4. I don’t just watch videos on SFV platforms; I also watch live streams and other content.	[75]
Social Interaction (SI)	SI1. I like to forward SFVs to family and friends. SI2. This platform allows me to form friendly relationships with other users. SI3. Sellers on the platform respond to my questions in a timely manner. SI4. I will often give reviews of items on SFV platforms.	[76]
Ease of Use (EU)	EU1. I think SFV platform has easy access to product information and I can easily find the desired products. EU2. I think the simplicity of the SFV platform will make product information more accessible. EU3. I think it’s easier to shop on SFV platforms. EU4. I think watching SFVs is more efficient than viewing images.	[77]
Recommendation Affordance (RA)	RA1. SFV platforms can provide personalized contents or products for my personal needs. RA2. SFV platforms can focus on my needs for products or services. RA3. The contents or products recommended by the SFV platform are very appealing to me RA4. SFV platforms can provide me with the kind of videos that I might like.	[48,78]
Social Belonging (SB)	SB1. I think I am a bit similar to other consumers of the SFV platform in some ways. SB2. I have an inexplicable affinity with people who shop on the same SFV platforms. SB3. After interacting with other users, I would like to purchase a product/service from this platform. SB4. If there is a product I would like to purchase, I would like to communicate with other users on this platform.	[79]
Perceived Trust (PT)	PT1. I am sure the SFV platforms won’t let unscrupulous merchants get past the vetting process. PT2. I believe the SFV platform will recommend us good products. PT3. I believe that the SFV platform will not post false information to deceive consumers. PT4. I believe that the information I get on SFV platforms is true.	[55,80]
Product Relevance (PR)	PR1. The advertised products might be of value to me. PR2. The advertised products are relevant to my needs. PR3. The advertised product is the one I am looking for. PR4. The advertised videos spoke to my concerns.	[58]
